# Latent anxiety and depression dimensions differ amongst patients with eating disorders: A Swedish nationwide investigation

**DOI:** 10.1002/mpr.1961

**Published:** 2023-02-12

**Authors:** Christopher Hübel, Andreas Birgegård, Therese Johansson, Liselotte V. Petersen, Rasmus Isomaa, Moritz Herle

**Affiliations:** ^1^ Social, Genetic & Developmental Psychiatry Centre Institute of Psychiatry, Psychology and Neuroscience King's College London London UK; ^2^ UK National Institute for Health Research (NIHR) Biomedical Research Centre for Mental Health South London and Maudsley Hospital London UK; ^3^ National Centre for Register‐based Research Aarhus BSS Business and Social Sciences Aarhus University Aarhus Denmark; ^4^ Department of Pediatric Neurology Charité Universitätsmedizin Berlin Berlin Germany; ^5^ Department of Medical Epidemiology and Biostatistics Karolinska Institutet Stockholm Sweden; ^6^ Department of Immunology, Genetics and Pathology Science for Life Laboratory Uppsala University Uppsala Sweden; ^7^ Centre for Women's Mental Health During the Reproductive Lifespan—Womher Uppsala University Uppsala Sweden; ^8^ The Wellbeing Services County of Ostrobothnia Abo Finland; ^9^ Faculty of Education and Welfare Studies Åbo Akademi University Vasa Finland; ^10^ Department of Biostatistics & Health Informatics Institute of Psychiatry, Psychology & Neuroscience King's College London London UK

**Keywords:** anorexia nervosa, binge‐eating disorder, bulimia nervosa, disinterest, factor analysis, psychometrics, questionnaire

## Abstract

**Objective:**

Anxiety and depression symptoms are common in individuals with eating disorders. To study these co‐occurrences, we need high‐quality self‐report questionnaires. The 19‐item self‐rated Comprehensive Psychopathological Rating Scale for Affective Syndromes (CPRS‐S‐A) is not validated in patients with eating disorders. We tested its factor structure, invariance, and differences in its latent dimensions.

**Method:**

Patients were registered by 45 treatment units in the Swedish nationwide Stepwise quality assurance database for specialised eating disorder care (*n* = 9509). Patients self‐reported their anxiety and depression symptoms on the CPRS‐S‐A. Analyses included exploratory and confirmatory factor analyses (CFA) in split samples, and testing of invariance and differences in subscales across eating disorder types.

**Results:**

Results suggested a four‐factor solution: Depression, Somatic and fear symptoms, Disinterest, and Worry. Multigroup CFA indicated an invariant factor structure. We detected the following differences: Patients with anorexia nervosa binge‐eating/purging subtype scored the highest and patients with unspecified feeding and eating disorders the lowest on all subscales. Patients with anorexia nervosa or purging disorder show more somatic and fear symptoms than individuals with either bulimia nervosa or binge‐eating disorder.

**Conclusion:**

Our four‐factor solution of the CPRS‐S‐A is suitable for patients with eating disorders and may help to identify differences in anxiety and depression dimensions amongst patients with eating disorders.

## 1 INTRODUCTION

### 1.1 Comorbidity amongst affective disorders and eating disorders

Symptoms of anxiety and depression, such as disinterest, low mood, and suicidality are commonly found amongst patients with eating disorders in the population and clinic (Dolan et al., 2021; Eckert et al., 1982; Kaye et al., 2004; Martín et al., 2019; Puccio et al., 2016; Udo & Grilo, 2019). Comorbid depression amongst individuals with any eating disorders is highly prevalent with 75% (Godart et al., 2015). Specifically, ∼60% of adolescents with anorexia nervosa report depressive symptoms (Blinder et al., 2006; Bühren et al., 2014) and patients with anorexia nervosa often have comorbid clinical depression (Jaite et al., 2013; Ulfvebrand et al., 2015). This high level of comorbidity with depression is also observed in individuals with bulimia nervosa, in population (Hudson et al., 2007; Swanson et al., 2011) and clinical samples (Fischer & le Grange, 2007; Kaye et al., 2004; Ulfvebrand et al., 2015). Similarly to depression, anxiety and eating disorders co‐occur (Garcia et al., 2020; Godart et al., 2002; Kaye et al., 2004; Kerr‐Gaffney et al., 2018; Steinhausen et al., 2015; Swinbourne et al., 2012; Ulfvebrand et al., 2015). However, prevalence estimates show considerable heterogeneity depending on measure or assessment type (e.g., self‐report vs. clinical interview; Godart et al., 2007; Meier et al., 2015). Overall, the co‐occurrence of depressive or anxiety symptoms with eating disorder symptoms complicates treatment (Brand‐Gothelf et al., 2014; Martín et al., 2019; Thornton et al., 2011). Therefore, accurate assessment of anxiety and depression symptoms in eating disorders may be beneficial for treatment planning.

### 1.2 Measurement issues

To study co‐occurring symptoms among eating disorders, anxiety, and depression, we need high‐quality measures of symptoms to delineate differences in eating disorder presentation in clinical and population samples. To assesses anxiety and depression symptoms, measures like the Patient Health Questionnaire‐9 (PHQ‐9; Kroenke et al., 2001) the Generalised Anxiety Disorder Assessment (GAD‐7; Spitzer et al., 2006), the Symptom Checklist‐90 (SCL‐90; Fittig et al., 2008) and its short form, the Brief Symptom Inventory (BSI; Beesdo‐Baum et al., 2012) are widely used in research. The PHQ‐9 and GAD‐7 factor structures and scores have been validated in samples of patients with eating disorders and the general population, indicating that both questionnaires are suitable (Wisting et al., 2021). However, as PHQ‐9 and GAD‐7 are strictly based on diagnostic criteria, these questionnaires only cover a limited range of anxiety and depression symptoms. The SCL‐90 and BSI cover a wider range of symptoms but are less frequently used in eating disorder research. Additionally, studies often only calculate global summary scores, ignoring more fine‐grained information of subscales. Hence, in order to explore the whole spectrum of symptoms, broader assessment tools to better understand the heterogeneity in eating disorder presentations, evading the cost‐ and time‐related limitations of diagnostic interviews, are urgently needed. One potential scale of interest is the Comprehensive Psychopathological Rating Scale (CPRS; Asberg & Schalling, 1979) which consists of 65 items and was originally developed to evaluate treatment outcomes in psychological interventions. The scale includes items covering symptoms of psychiatric disorders, such as schizophrenia but also anxiety and depression. The scale was originally developed in Sweden, and has been translated into most other European languages. The complete version of the CPRS is rarely used, but shorter subscales have been deemed to be more useful, such as the Montgomery Åsberg Depression Rating Scale (MADRS; Montgomery & Asberg, 1979) and the Self‐rating Scale for Affective Syndromes (CPRS‐S‐A; Svanborg & Asberg, 1994). The latter is in focus here, and is designed to contain subscales for depression, anxiety, and compulsivity.

A previous analysis of the CPRS‐S‐A questionnaire in a subsample of the data available for our investigation compared a global CPRS‐S‐A score across patients with different eating disorders. Results showed that patients with an unspecified feeding or eating disorder reported fewer problems than patients with other eating disorders. Additionally, patients with the anorexia nervosa binge‐eating/purging subtype reported more problems compared with atypical anorexia nervosa patients (Ekeroth et al., 2013). One issue of the questionnaire is the construction of its three subscales. When calculating the subscales, it is advised to include the same item in several subscales. Therefore, the subscales are highly correlated. In the previous analyses the correlations ranged from 0.78 to 0.86 and are inflated, rendering the original subscales unreliable. Therefore, in this study, we investigated differences in depression and anxiety dimension amongst patients with eating disorders using newly derived subscales of the Self‐rating Scale for Affective Syndromes (CPRS‐S‐A); a short form of the CPRS (Svanborg & Asberg, 1994) in one of the world's largest clinical sample of more than 9000 patients with eating disorders in Sweden.

## 2 METHODS

### 2.1 Sample

The sample comprises inpatients and outpatients registered by 45 treatment units in the Stepwise quality assurance database for specialised eating disorder care in Sweden aged 18 years and older (Birgegård et al., 2010). The 45 treatment units comprise eating disorder clinics, units, or teams of different sizes. Depending on the time period, up to 10 units have had specialised inpatient services in‐house. All units have access to psychiatric (most often) or somatic inpatient services within their clinics or at the nearest hospital if it is a smaller team or unit. Sizes of the units vary greatly from Stockholms centrum för ätstörningar (SCÄ) with ∼100 staff and 1000–1200 new patients/year to teams of two people. Six units are associated with university hospitals. Stepwise is a nationwide internet‐based data collection system, which includes individuals through medical or self‐referral, if intention to treat has been established, and if the individual received a formal eating disorder diagnosis (Birgegård et al., 2022). The database has been used since 2005 and our data were extracted on November 23, 2017. At data extraction, approximately 10,470 adult entries had been registered (Supplementary Table S1).

### 2.2 Eating disorder diagnosis

Clinicians registered patients' eating disorders diagnosis based on Diagnostic and Statistical Manual of Mental Disorders, Fourth Edition (DSM‐IV; American Psychiatric Association, 2013; Birgegård et al., 2022). In our analysis, we translated DSM‐IV to DSM‐5 eating disorders to reflect the current understanding of eating disorders. Depending on the patient's endorsement of binge eating or purging in either the Eating Disorder Examination questionnaire (Luce & Crowther, 1999) or the Structured Eating Disorder Interview (de Man Lapidoth & Birgegård, 2010), we re‐assigned DSM‐5 diagnoses. We used 18.5 kg/m^2^ as the cutoff value for underweight in anorexia nervosa. Anorexia nervosa without weight criterion (*n* = 50) or without amenorrhea (*n* = 186) that had a body mass index (BMI) lower than 18.5 kg/m^2^ who endorsed any binge eating or purging were assigned anorexia nervosa binge‐eating/purging. If none endorsed, they were assigned an anorexia nervosa restricting diagnosis (*n*_without weight criterion_ = 84 or *n*_without amenorrhea_ = 144). If their BMI was above 18.5 kg/m^2^, we assigned an atypical anorexia nervosa diagnosis (*n*_without weight criterion_ = 441 or *n*_without amenorrhea_ = 402). Eating Disorder Not Otherwise Specified type 3 or bulimia nervosa without sufficient duration/frequency criteria (*n* = 833) was assigned as bulimia nervosa diagnosis because those criteria are relaxed in DSM‐5. Further, Eating Disorder Not Otherwise Specified example 4 was kept as ‘purging disorder’. The remaining unspecified eating disorders that were not classified into either of these categories were termed ‘unspecified feeding or eating disorder’ (UFED), consisting of patients with ‘chewing and spitting’, bulimia nervosa/binge‐eating disorder with low frequency/duration, or other residual types that did not fit any of the main categories (Supplementary Table S1).

### 2.3 Exclusion

We excluded 801 duplicated entries of repeated registrations of the same individual, keeping the first registration. Subsequently, we iteratively excluded two individuals with missing age, 16 not assigned a treatment centre, 120 without a clinical eating disorder diagnosis, and 22 because they had not answered the CPRS questionnaire. The final sample comprised 9509 patients with eating disorders.

### 2.4 Ethics

When patients were entered into the database, clinicians recorded consent for general research use of their data and 3% declined participation. This study is approved by the Stockholm Regional Ethics Board (Reg. no. 2009/196‐31/4).

### 2.5 Comprehensive Psychopathological Rating Scale, self‐rated version for Affective Syndromes

At registration, the patients answered 19 items of the CPRS‐S‐A. We present the instrument as Supplementary Material. The answer options are different for each question, but they are on a scale from 0 to 3, rated in 0.5‐point increments. We recoded these values to 0–6. We renamed item 19, titled ‘Zest for life’ in the MADRS‐S to ‘Suicidal thoughts’ to represent its content better.

### 2.6 Exploratory factor analyses

**FIGURE 1:**
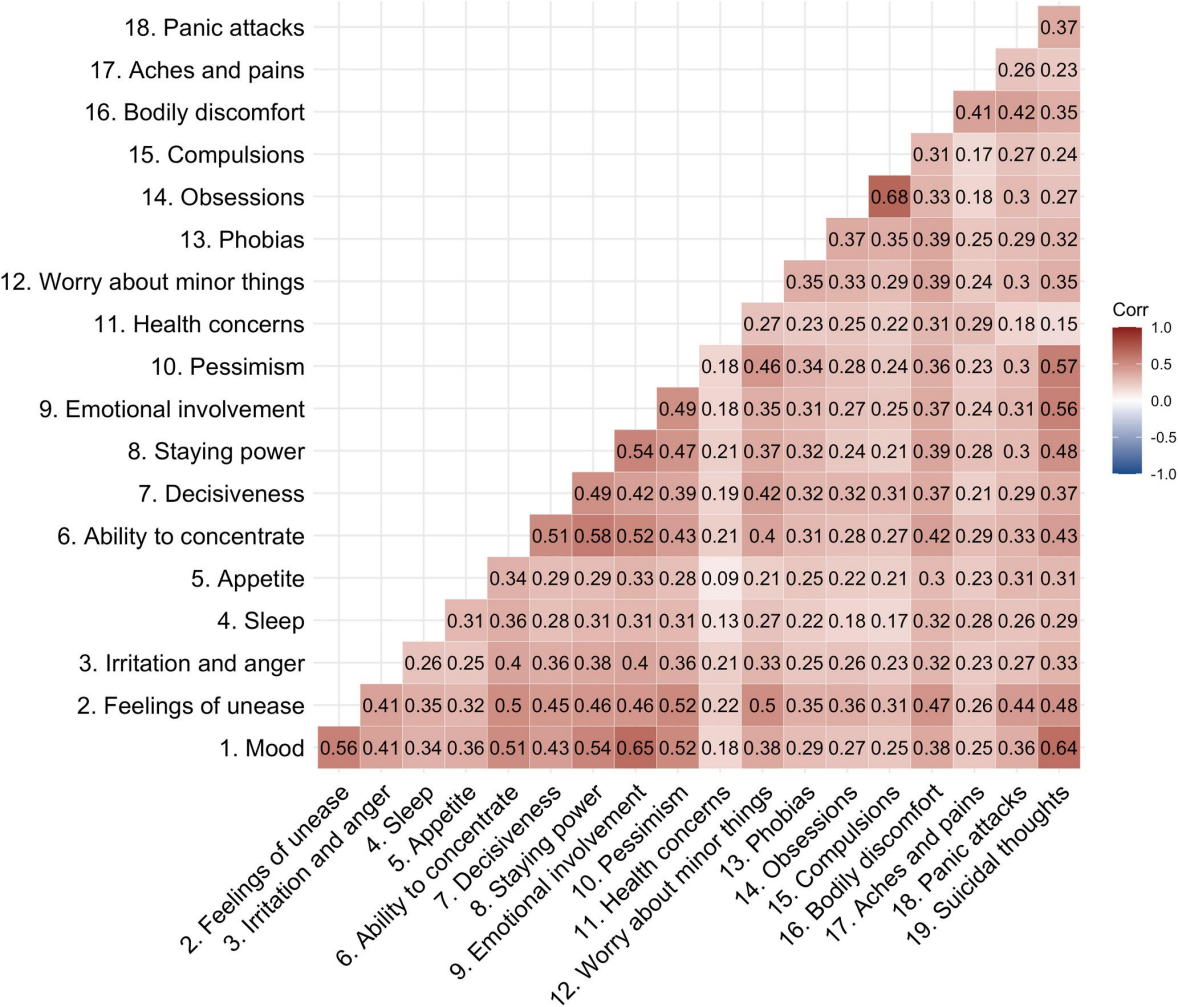
Pairwise Pearson's correlations amongst the Self‐rating Scale for Affective Syndromes (CPRS‐S‐A) items. We calculated the correlations in 9509 participants registered in Stepwise, the Swedish clinical eating disorder database. We estimated the number of independent traits in the matrix using the Galwey method and adjusted the *α* threshold (*α* = 0.003) accordingly. All correlations are statistically significant at this *α* threshold. Saturation represents the strength of the correlation. Positive correlations are red.

We calculated pairwise Pearson correlations amongst all items (Figure 1) in the full sample (*n* = 9509). We inspected the matrix visually for singularity, multicollinearity, and redundancy of items (i.e., values <0.30 and >0.90). We calculated the determinant of the matrix (Dziuban & Shirkey, 1974), the Kaiser‐Meyer‐Olkin (KMO) statistic (Kaiser, 1974), and performed Bartlett's Test of Sphericity (Bartlett, 1950), to test if our data are suitable for an exploratory factor analysis. To inform our decision on the underlying factor structure, we performed parallel analysis (Horn, 1965), and calculated the Very Simple Structure criterion (VSS; Revelle & Rocklin, 1979), and Velicer's Minimum Average Partial criterion (Velicer, 1976). We performed the exploratory factor analysis on 70% (*n* = 6656) of the sample using the maximum likelihood estimator in the ‘psych’ R package (Revelle & Revelle, 2015). Given that the CPRS items have seven answer options, we treated them as continuous. We allowed the factors to correlate using oblimin rotation. To judge the fit of our model, we applied the criteria as outlined in Table 1 (Hu & Bentler, 1999). We retained the items with factor loadings of >0.30. If multiple models showed adequate fit, we would choose the model with factors that encompass the greatest number of items.

**TABLE 1:** Criteria for a good fit (Hu & Bentler, 1999)

Root mean square error of approximation (RMSEA)	≤0.05
Tucker Lewis index (TLI)	≥0.95
Standardised root mean square residuals (SRMR)	≤0.05
Bayesian information criteria (BIC)	Smaller than other models

### 2.7 Confirmatory factor analysis and factor scores

We validated our exploratory factor analysis model with a confirmatory factor analysis (CFA) on the remaining 30% participants using the ‘lavaan’ R package (Rosseel, 2012). We interpreted fit statistics (Hu & Bentler, 1999; Schreiber et al., 2006) and considered a Comparative Fit Index (CFI) ≥0.95 as good fit. Subsequently, we computed the CFA in the full sample (*n* = 9509) to provide fit statistics and calculate factor scores, using the Bartlett estimator for continuous items.

### 2.8 Descriptive indices and psychometric properties

**FIGURE 2:**
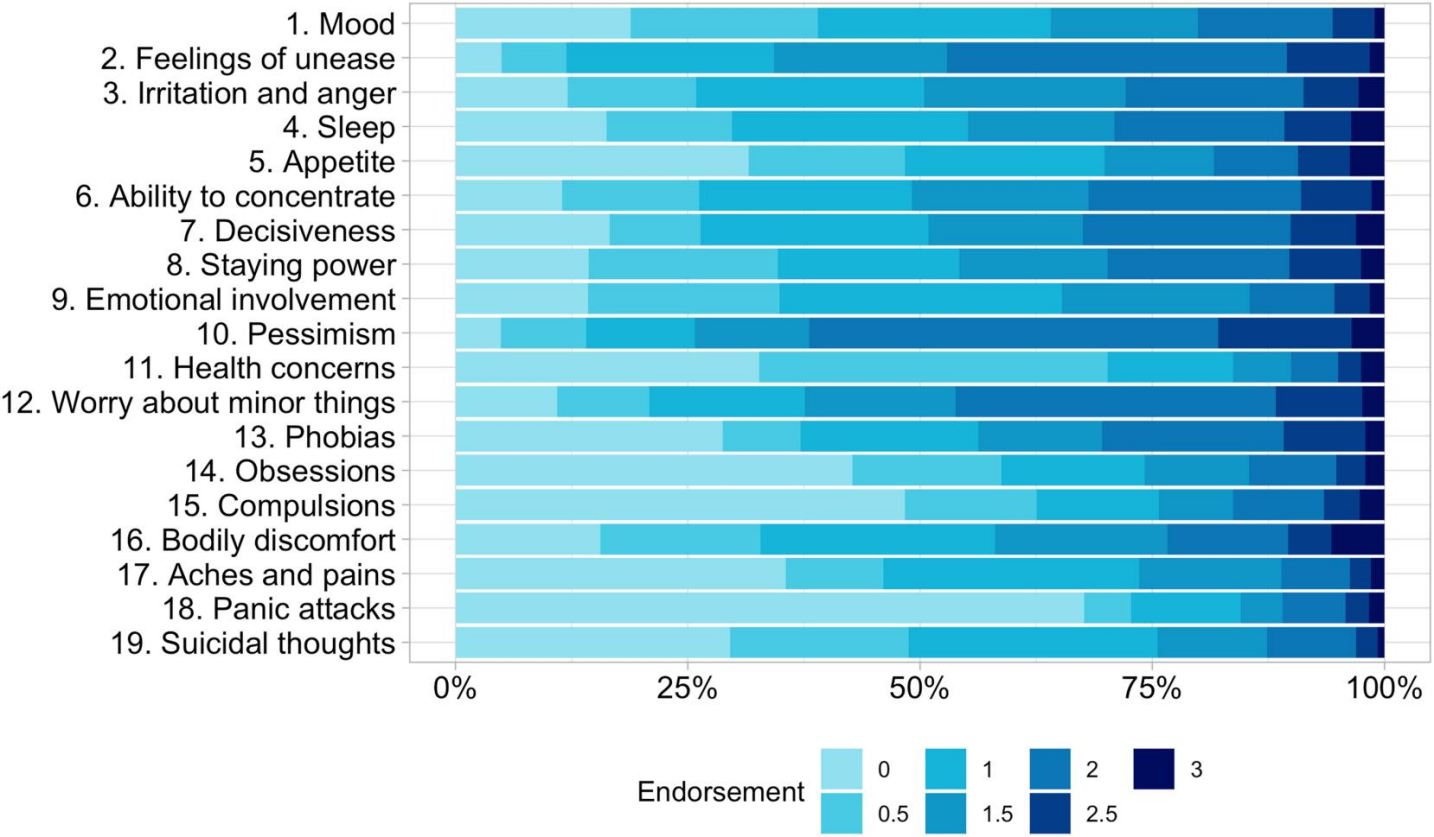
Endorsement of the Comprehensive Psychopathological Rating Scale Self‐rating Scale for Affective Syndromes (CPRS‐S‐A) 19 items version in the Stepwise sample (*n* = 9509). The saturation of blue indicates a higher endorsement on the specific item. We display percentages. The answer options differed across items, with higher values indicating a stronger endorsement.

We show responses to the individual items as frequency plots (Figure 2) and distributions of the factor scores as histograms and qq plots for the complete sample (Supplementary Figure S1) while presenting box plots per eating disorder (Figure 4). We also report mean and standard deviations for our generated factor scores and report Cronbach's *α* (Bland & Altman, 1997; Cronbach, 1951) and McDonald's *ω* (Hayes & Coutts, 2020) as measures of internal consistency.

### 2.9 Multigroup confirmatory factor analysis

We performed a multigroup confirmatory factor analysis (MGCFA) to test if the questionnaire elicits the same responses, response patterns, and has the same underlying factor structure across eating disorder diagnostic groups. If statistical invariance in responding is found, then we can compare scores and subscale scores across groups. Different types of measurement invariance exist: configural, the factor structure is similar across groups; metric, factor loadings are similar across groups; scalar, intercepts (i.e., group means) are similar; and strict, residuals (i.e., variances) are similar across the groups. We tested for these invariance models in a stepwise procedure from the least restricted model to the fully restricted model. Overall, invariance indicates that different groups are from the same population.

### 2.10 Group comparisons

We judged the distribution of the factor scores by visually inspecting qq and distribution plots (Supplementary Figure S1). None of the four subscales showed a normal distribution. Therefore, we performed non‐parametric Kruskal‐Wallis one‐way ANOVAs. If significant, Dunn's post‐hoc tests were carried out with a Benjamini Hochberg‐adjusted level of significance for the pairwise comparisons.

### 2.11 Convergent and divergent validity

If our newly developed dimensions correlate with other instruments that measure similar constructs in the expected direction and with sufficient magnitude, they show convergent validity. To assess this, we estimated correlations with the original depression and anxiety subscales of the CPRS‐S‐A, the Clinical Impairment Assessment (CIA) total score (Bohn et al., 2008; both expected to be positive), and the Structural Analysis of Social Behaviour (SASB) self‐affirmation scale (expected to be negative; Benjamin, 1974). For divergent validity, we correlated the new CPRS‐S‐A dimensions with variables that we expected to be unrelated: SASB self‐control scale and height.

## 3 RESULTS

### 3.1 Descriptives

The patients in our sample were on average 26 years (SD = 8) old and the age ranged from 18 to 70 years, with 96% of the sample being female. Of the patients, 1363 (14%) received an anorexia nervosa restricting, 702 (7%) anorexia nervosa binge‐eating/purging, 832 (9%) an atypical anorexia nervosa, 3,807 (40%) a bulimia nervosa, 658 (7%) a binge‐eating disorder, 1711 (18%) a purging disorder, and 436 an unspecified feeding and eating disorders diagnosis (5%; Supplementary Table S1).

### 3.2 Descriptives of the CPRS‐S‐A in stepwise

The CPRS‐S‐A showed a Cronbach's *α* (*α* = 0.90) and McDonald's *ω* (*ω* = 0.92) in our sample (Supplementary Table S2). The distributions of answers to the questionnaire items are displayed in Figure 2 for the full sample and Supplementary Table S3 for the discovery sample.

### 3.3 Suitability of the data for factor analysis

Prior to factor analyses, the suitability of the data was investigated. None of the items showed zero or near‐zero variance (Supplementary Table S4). Kaiser‐Meyer‐Olkin measure (KMO = 0.94, Supplementary Table S5) and significant Bartlett test of sphericity (*p* < 2.22 × 10^−16^) indicated that the data were suitable for factor analyses. Pearson's correlations ranged from 0.09 to 0.68 (Figure 1). The exploratory factor analysis was conducted on one random split of the sample (*n* = 6656; 70%). As we were primarily interested in core anxiety and depression symptoms, we excluded the items ‘14. Obsessions’ and ‘15. Compulsions’ from the factor analysis. Furthermore, they loaded strongly on one factor by themselves, representing an index of compulsion. If these items had remained in the model, they would have lowered our power to measure meaningful underlying factors as they would have distorted the model towards their own factor. We, furthermore, excluded the item ‘11. Health concerns’, because its correlation with the other items was small (*r* = 0.09–0.29; Figure 1), rendering it unsuitable for factor analysis. Cronbach's *α* remained stable after these items were dropped (Supplementary Table S6).

### 3.4 Exploratory factor analysis

**FIGURE 3:**
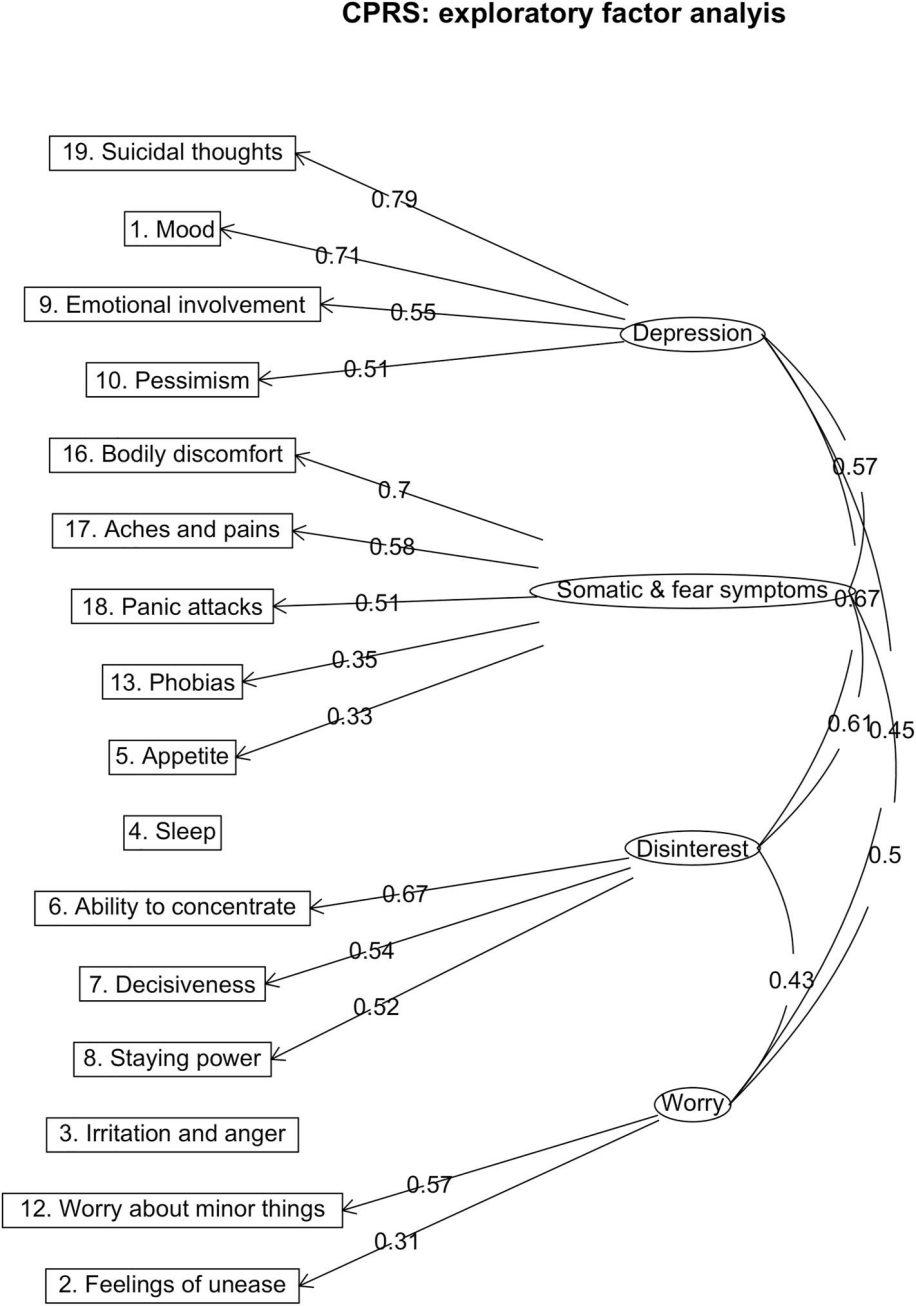
Exploratory factor analysis of 16 items of the Comprehensive Psychopathological Rating Scale Self‐rating Scale for Affective Syndromes (CPRS‐S‐A). The path diagram shows item factor loadings and between‐factor correlations for the four factors of Depression, Somatic and fear symptoms, Disinterest, and Worry. Paths with a factor loading of <0.3 were omitted.

Very simple structure (Supplementary Table S7) and parallel analysis (Supplementary Table S8) suggested a one‐factor solution. However, as we are interested in different anxiety and depression dimensions, a comparison of fit statistics suggested that the five‐factor solution fitted the data best. However, the model contained two factors on which only one item loaded (i.e., 2. Feelings of unease and 12. Worry about minor things) and therefore the model was deemed unsuitable. Hence, we chose the four‐factor solution as our final model which explained 34% of the total variance. The factor solution had a low RMSEA (0.042, 90% CI: 0.039 0.045) and low Bayesian Information Criterion (BIC = 245; for full results, see Table 2 and Supplementary Tables S9–13). As factors were considered to be correlated, factors were realigned using an oblique rotation. The factor loadings for each item, after rotation, are listed in Figure 3. Items 3 and 4 (Irritation and anger, and Sleep, respectively) did not load on any of the factors and are therefore not included in the confirmatory factor analysis. We labelled the four factors: F1 Depression, F2 Somatic and fear symptoms, F3 Disinterest, and F4 Worry.

**TABLE 2:** Model fit statistics for exploratory factor analysis

Number of factors	df	RMSEA (≤0.05)	RMSEA 90% CI	TLI (≥0.95)	BIC	SRMR (≤0.05)	Cumulative variance	Minimum item loading
1	104	0.078	[0.076, 0.080]	0.882	3351	0.05	0.38	16
2	89	0.062	[0.060, 0.065]	0.923	1608	0.03	0.35	7
3	75	0.053	[0.051 0.056]	0.944	840	0.03	0.33	3
**4**	**62**	**0.042**	**[0.039 0.045]**	**0.965**	**245**	**0.02**	**0.34**	**2**
5	50	0.035	[0.033 0.038]	0.975	29	0.01	0.34	1

*Note*: The cut off for each statistic to signify good fit is listed in each header (Hu & Bentler, 1999). The model with the lowest BIC is preferred, Cumulative variance indicates the part of the total variance explained by all items comprising the factors. The factor analysis was performed on 16 items of the Comprehensive Psychopathological Rating Scale Self‐rating Scale for Affective Syndromes (CPRS‐S‐A) in the Swedish quality register for eating disorder care, Stepwise (*n* = 6656). Model in bold was chosen as the best fitting model.

Abbreviations: BIC, Bayesian Information Criterion; df, Degrees of freedom; RMSEA, Root Mean Square Error of Approximation; SRMR, Standardised Root Mean Square Residuals; TLI, Tucker‐Lewis Fit Index.

### 3.5 Confirmatory factor analysis

**FIGURE 4:**
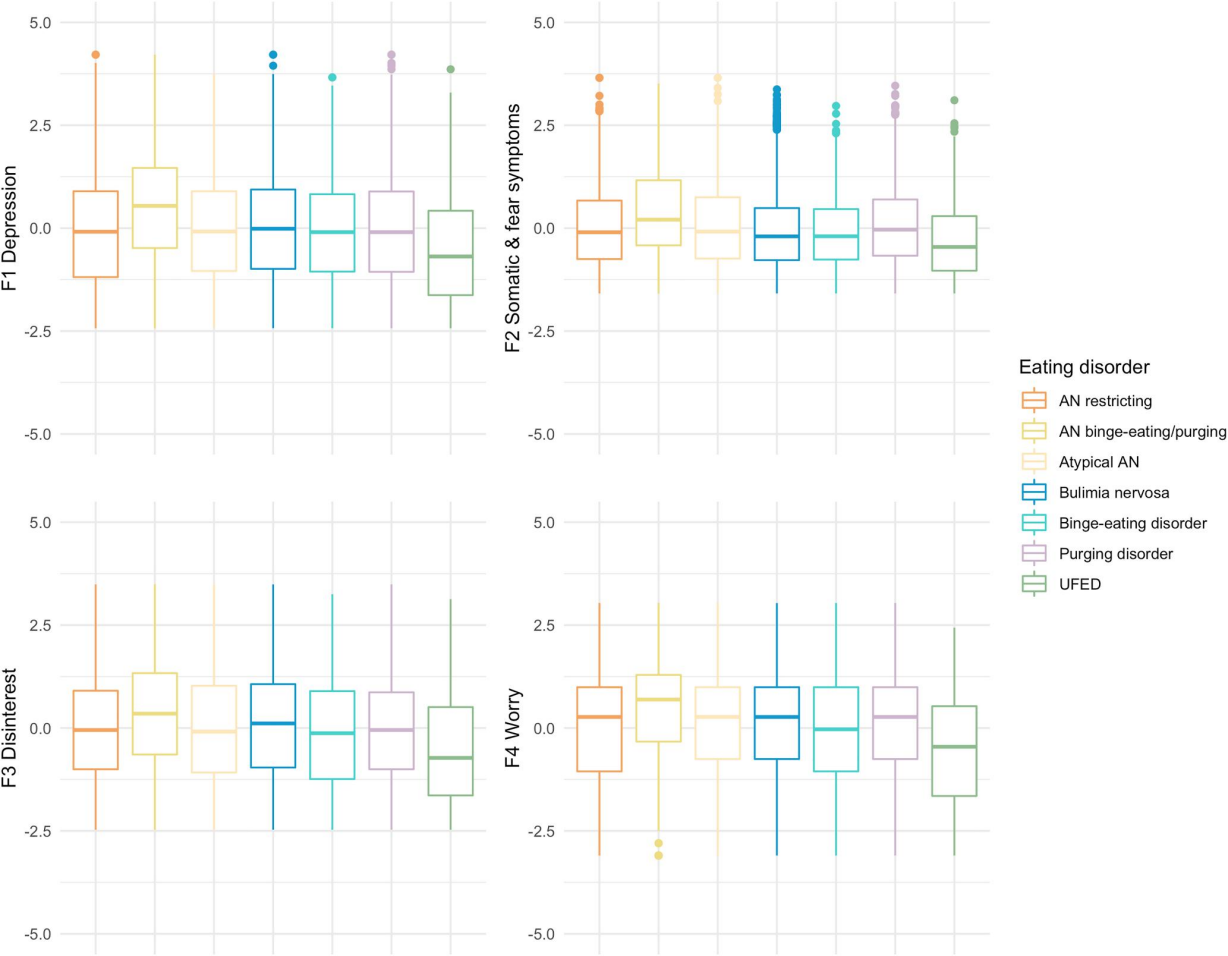
Distribution of factor scores across eating disorder types. Boxplots represent median and interquartile range in the whole Stepwise sample (*n* = 9509). AN, anorexia nervosa, UFED, unspecified feeding or eating disorder.

We conducted the CFA in the remaining 30% of the sample (*n* = 2853; Supplementary Table S14). Results confirmed the four‐factor model. The RMSEA (0.060, 90% CI: 0.056, 0.064), the CFI (0.952), and the SRMR (0.032) indicated good model fit (Hu & Bentler, 1999). The TLI (0.939) was slightly above the standard threshold. We also ran the confirmatory factor analysis in the full sample (*n* = 9509) which yielded the following fit statistics: CFI = 0.953, TLI = 0.940, RMSEA = 0.060 [90% CI, 0.058, 0.062], SRMR = 0.030). We show the resulting factor scores and their distribution in Figure 4 and Supplementary Figure S1.

### 3.6 Multigroup confirmatory factor analysis

Our multigroup confirmatory factor analysis resulted in full configural and metric invariance, indicating that the factor structure and the factor loadings are comparable across eating disorders (Supplementary Table S15). Furthermore, the questionnaire showed partial scalar invariance when freeing up the intercepts of item five and eight, meaning that the means were similar across groups apart from item five (less appetite) and eight (less motivation).

### 3.7 Factor scores

We calculated factor scores for each individual based on the final model (Figure 4 and Supplementary Table S16). We compared the factor scores using Kruskal–Wallis one‐way analysis of variance, and Dunn's post hoc test (Table 3). Overall, individuals with anorexia nervosa binge‐eating/purging scored higher on all four subscales than all other eating disorders, including the restricting subtype of anorexia nervosa. However, there was no statistically significant difference on any of the four scores between the restricting subtype and atypical anorexia nervosa or purging disorder. Individuals with unspecified feeding and eating disorders scored lower on all four scales than all other eating disorders.

**TABLE 3:** Median differences and results from Dunn's post‐hoc tests

Factor	ANR versus ANBP	ANR versus AAN	ANR versus BN	ANR versus BED	ANR versus PUR	ANR versus UFED	ANBP versus AAN	ANBP versus BN	ANBP versus BED	ANBP versus PUR	ANBP versus UFED	AAN versus BN	AAN versus BED	AAN versus PUR	AAN versus UFED	BN versus BED	BN versus PUR	BN versus UFED	BED versus PUR	BED versus UFED	PUR versus UFED
F1	0.6****	0.0	0.1	0.0	0.0	−0.6****	−0.6****	−0.5****	−0.6****	−0.6****	−1.2****	0.1	0.0	0.0	−0.6****	−0.1	−0.1	−0.7****	0.0	−0.6****	−0.6****
F2	0.3****	0.0	−0.1**	−0.1	0.1	−0.4****	−0.3****	−0.4****	−0.4****	−0.2****	−0.7****	−0.1***	−0.1**	−0.1**	−0.4****	0.0	0.2****	−0.3****	0.2**	−0.3***	−0.5****
F3	0.3****	−0.1	0.1*	−0.1	0.0	−0.7****	−0.4****	−0.2****	−0.4****	−0.3****	−1.0****	0.2	0.0	0.0	−0.6****	−0.2**	−0.1**	−0.8****	0.1	−0.6****	−0.7****
F4	0.4****	0.0	0.0	−0.3*	0.0	−0.8****	−0.4****	−0.4****	−0.7****	−0.4****	−1.2****	0.0	−0.3*	−0.3*	−0.8****	−0.3**	0.0	−0.8****	0.3**	−0.5****	−0.8****

*Note*: We performed Kruskal‐Wallis one‐way Analysis of Variance (ANOVA) and Dunn's post‐hoc tests for pairwise comparisons and judged significance by adjusting the alpha level using the Benjamini Hochberg approach. F1 Depression, F2 Somatic and fear symptoms, F3 Disinterest, and F4 Worry.

Abbreviations: AAN, atypical anorexia nervosa; ANBP, anorexia nervosa binge‐eating/purging; ANR, anorexia nervosa restricting; BED, binge‐eating disorder; BN, bulimia nervosa; PUR, purging disorder patients; UFED, unspecified feeding or eating disorder.

**** <1 × 10^−4^, *** <0.001, ** <0.01, * <0.05.

On factor 2 Somatic and fear symptoms, patients with anorexia nervosa or purging disorder scored higher than individuals with either bulimia nervosa or binge‐eating disorder. Patients with anorexia nervosa restricting subtype or atypical anorexia nervosa reported depressive symptoms on the same median level as patients with either bulimia nervosa, binge‐eating disorder, or purging disorder. However, compared with anorexia nervosa binge‐eating/purging, all other eating disorders reported fewer depressive symptoms. On factor 3 Disinterest, the results showed a mixed picture: patients with either anorexia nervosa binge‐eating/purging subtype or bulimia nervosa reported more disinterest than the other eating disorders.

### 3.8 Convergent and divergent validity

**FIGURE 5:**
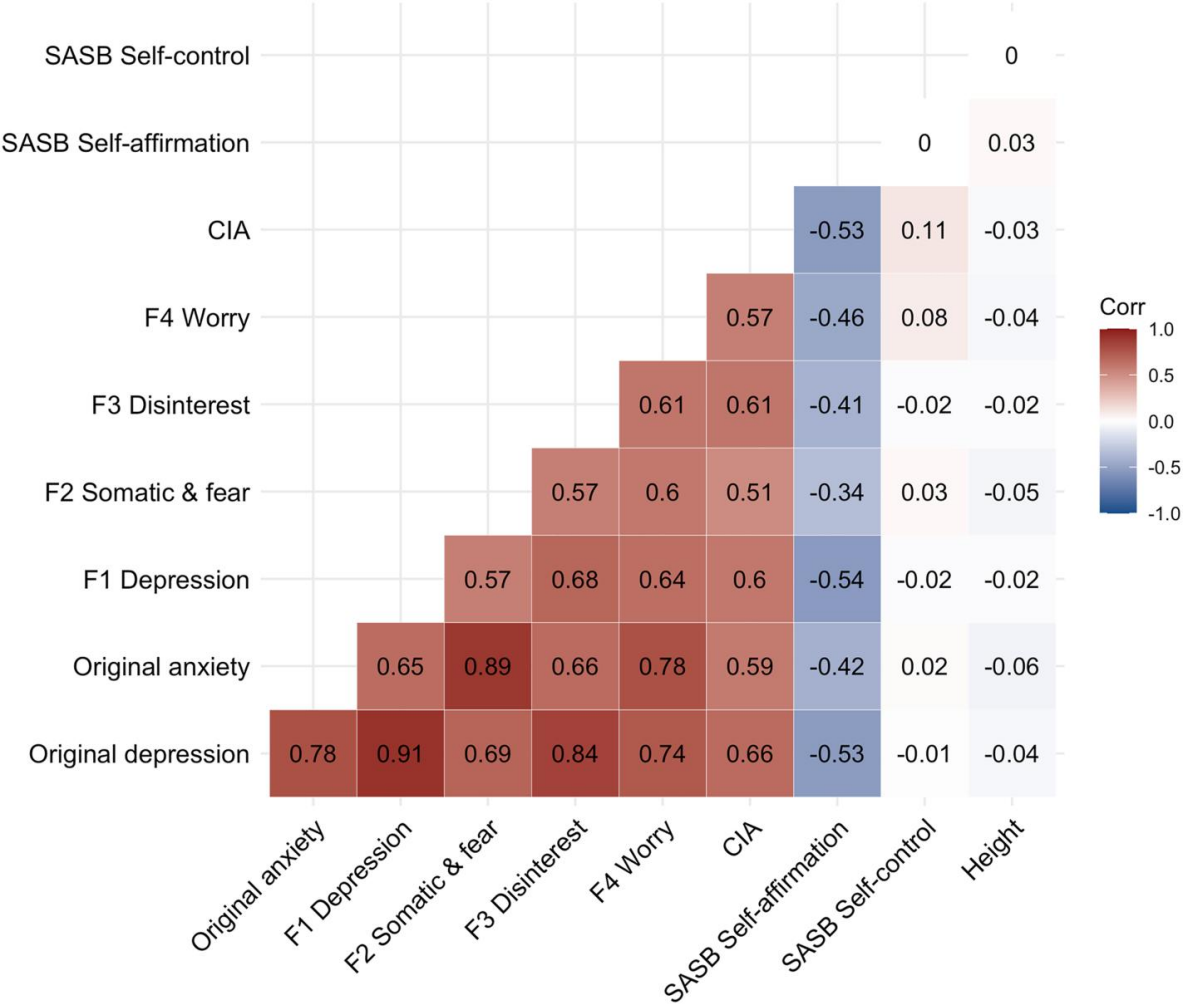
Correlation matrix of CPRS‐S‐A dimensions with external correlates. The correlation matrix shows Pearson's correlations of the original CPRS‐S‐A anxiety and depression dimensions, the newly derived 4 factor solution, the Clinical Impairment Assessment (CIA) total score, and both subscales of the Structural Analysis of Social Behaviour (SASB) self‐affirmation and self‐control als well as height. Sample sizes range between 8146 and 9509. Positive correlations are red and negative correlations are blue.

Correlations between the original CPRS subscales and the new factors were positive and high (range *r* = 0.65–0.91), indicating that they measure similar underlying constructs (Figure 5). Importantly, the correlations between the new dimensions (range *r* = 0.51–0.68) were lower than between the original CPRS scales of depression and anxiety (*r* = 0.78), indicating that the new dimensions measure diverging underlying constructs. As hypothesised, the new CPRS dimensions were not correlated with either SASB self‐control (range *r* = −0.02–0.08) or height (range *r* = −0.05 to −0.02), but were positively correlated with the CIA total score (range *r* = 0.51–0.61) and negatively with self‐affirmation (range *r* = −0.54 to −0.34).

## 4 DISCUSSION

### 4.1 Summary

**Summary of findings.** Using a factor analytic approach, we propose and confirm a four‐factor structure of the Self‐rating Scale for Affective Syndromes (CPRS‐S‐A) in the world's largest clinical sample of 9509 patients with eating disorders. The four dimensions capture specific aspects of depression and anxiety: 1. Depression (4 items), 2. Somatic and fear symptoms (5 items), 3. Disinterest (3 items) and 4. Worry (2 items). Furthermore, we show that the new subscales can be used to measure differences in these dimensions amongst patients with eating disorders.

**Reduced scale.** Our psychometric analysis suggests items can be removed due to their low correlations with other items. Our proposed reduced scale has a total of 14 questionnaire items and is hence shorter than the original CPRS‐S‐A with 19 items. First, we could drop item ‘11. Health concerns’ as it barely correlated with other items on the scale. Second, we dropped two items regarding compulsiveness (i.e., item 14 and 15) as these were not deemed core to depression and anxiety and they were highly correlated with each other. Further, two items (i.e., 4. Sleep and 3. Irritation and anger) did not sufficiently load onto any of our four factors and were hence dropped.

### 4.2 Context of existing literature

**Difference to original scale.** Our factor structure differs substantially from the original CPRS‐S‐A (Svanborg & Asberg, 1994). In contrast to the original structure (Supplementary Material), our new structure splits traditional depression symptoms into separate dimensions: depression and disinterest. Depression mostly included indicator items of low mood, pessimism, and lack of enjoyment, whereas Disinterest revolved around cognition, such as lack of concentration and decision making. Anxiety symptoms were also split into two factors. First, the Somatic and fear symptoms factor grouped together general pain, bodily discomfort, physical panic attack symptoms, such as heart palpitations and dizziness, with phobias which can present with somatic symptoms. Furthermore, the phobia item contains a specific example about mealtimes which may contribute to its loading on the Somatic and fear factor: ‘It can also be feeling uncomfortable in the company of others, at meals with and in similar situations.’ Especially, patients with eating disorders may experience fear‐related bodily discomfort during and prior to mealtimes (Lloyd et al., 2021). This factor also included an item probing differences in appetite. This item may be inappropriate in the context of eating disorders as changes in appetite can be a central symptom of eating disorders; however, the changes may be diametral depending on the eating disorder type. The appetite item loaded poorly on its factor and may be removed at the researcher's or clinician's discretion. Second, the Worry factor groups General worry about minor things and Feeling of unease together. This differs from the original CPRS‐S‐A that combined worry symptoms with the somatic and fear‐based symptoms. Overall, our analyses suggest a substantially different factor structure compared with the original.

**General differences amongst patients with eating disorders.** We explored differences in the new subscales amongst patients with eating disorders. On the one hand, comparisons suggested that patients with anorexia nervosa binge‐eating/purging score higher on all four subscales, consistent with the previous report based on a subsample of our analysis (Ulfvebrand et al., 2015). On the other hand, patients with unspecified feeding and eating disorders had the lowest scores across all four subscales in line with their subsyndromal expression of eating disorders.

**Specific differences.** In addition to these overarching differences, we detected differences for specific factors. On factor 2 Somatic and fear symptoms, patients with anorexia nervosa or purging disorder scored higher than individuals with either bulimia nervosa or binge‐eating disorder. These differences may indicate that the somatic complications seen in anorexia nervosa (Westmoreland et al., 2016) and purging disorder may be captured by items on this factor summarising somatic fear symptoms. Furthermore, patients with anorexia nervosa and purging disorders may perceive these somatic and fear symptoms more strongly than patients with bulimia nervosa or binge‐eating disorder. Fear has been proposed as a fundamental mechanism in the development of anorexia nervosa (Murray et al., 2018).

Depression and anxiety are risk factors for eating disorders (Meier et al., 2015; Steinhausen et al., 2015), but certain symptoms of anxiety or depression may represent somatic or psychiatric complications or sequelae of the eating disorder itself. However, in some cases, depressive and anxiety symptoms may be independent of the eating disorder. This underscores the importance of investigating anxiety and depression on the dimension or symptom level rather than using total scores.

### 4.3 Limitations

Our study may be biased due to limitations. The sample consisted predominantly of women which limits the ability to identify sex differences. Eating disorders are more commonly diagnosed amongst women, however, men are underrepresented in eating disorder research. This may be due to a lack of awareness and understanding for these disorders among the wider community and clinicians or may represent an underlying true sex difference. Our sample included Swedish treatment seeking patients of mostly white European ancestry limiting the generalisability of our findings. Furthermore, patients in healthcare registers may represent a more severe subpopulation of individuals with eating disorders. Hence, the factor structure and our observed differences amongst patients with eating disorders may not replicate across other ancestry or cultural groups or in individuals with less severe presentations. Our analyses were cross‐sectional and did not include a comparison group without eating disorders or any psychiatric disorder.

### 4.4 Future directions

To address a few of our limitations, future studies should confirm our newly detected factor structure in community samples, samples with other psychiatric disorders, and include a healthy comparison group. Optimally, researchers would collect repeated measures of the CPRS‐S‐A that would further our understanding of how these constructs develop over time and how levels of depression, disinterest, fear, and worry may change with treatment. Future studies could investigate clinical cut‐offs to measure comorbid depressive and anxiety disorders.

### 4.5 Conclusions

In summary, our four‐factor solution of the CPRS‐S‐A is suitable for adult patients with different eating disorders and identifies differences in anxiety and depression dimensions. An easily administered, reliable self‐report measure for the most common forms of co‐occurring anxiety and depression symptoms in eating disorders is clinically and for research important. The CPRS‐S‐A may aid the clinician in case formulation and treatment planning. It may also be relevant for the patient's own understanding of their situation. A discussion between patient and clinician, facilitated by the individual CPRS‐S‐A results, of depression and anxiety dimensions or symptoms in relation to eating disorder symptoms may improve therapeutic alliance and thus treatment outcome.

## AUTHOR CONTRIBUTIONS

**Christopher Hübel**: Conceptualisation, Software, Visualisation, Formal analyses, Writing ‐ Original Draft, Writing ‐ Review & Editing. **Andreas Birgegård**: Data Curation, Supervision, Writing ‐ Review & Editing, Funding acquisition, Resources, **Therese Johansson**: Software, Writing ‐ Review & Editing. **Liselotte V. Petersen**: Supervision, Writing ‐ Review & Editing, Funding acquisition. **Rasmus Isomaa**: Supervision, Writing ‐ Review & Editing. **Moritz Herle**: Supervision, Conceptualisation, Writing ‐ Original Draft, Writing ‐ Review & Editing, Project administration.

## CONFLICT OF INTEREST

The authors report no conflict of interest.

## CODE AVAILABILITY

Code is available on https://github.com/topherhuebel/CPRS‐SA‐ED.

## Supporting information

Supporting Information S1

Supporting Information S2

## Data Availability

Due to regional legal regulations, the data from the quality register cannot be shared.
